# Genetic Variation and Breeding Signature in Mass Selection Lines of the Pacific Oyster (*Crassostrea gigas*) Assessed by SNP Markers

**DOI:** 10.1371/journal.pone.0150868

**Published:** 2016-03-08

**Authors:** Xiaoxiao Zhong, Dandan Feng, Hong Yu, Lingfeng Kong, Qi Li

**Affiliations:** Key Laboratory of Mariculture Ministry of Education, Ocean University of China, Qingdao, China; Aristotle University of Thessaloniki, GREECE

## Abstract

In breeding industries, a challenging problem is how to keep genetic diversity over generations. To investigate genetic variation and identify breeding signatures in mass selected lines of Pacific oyster (*Crassostrea gigas*), three sixth-generation selected lines and four wild populations were assessed using 103 single nucleotide polymorphism (SNP) markers. The genetic diversity data indicated that the selected lines exhibited a significant reduction in the observed heterozygosity and observed number of alleles per locus compared with the wild populations (*P*≤0.05), indicating the selected lines tended to lose genetic diversity contrasted with the wild populations. The unweighted pair-group method with arithmetic mean (UPGMA) analysis showed that the wild populations and selected lines were not separated into two groups. Using four outlier tests, a total of 17 loci were found under selection at two levels. The global outlier detection suggested that 4 common outlier loci were subject to selection using both the hierarchical island model and Bayesian likelihood approaches. At regional level, 3 SNPs were detected as outlier using at least two outlier tests and one outlier SNP (CgSNP309) was overlapped in the two wild-selected population comparisons. The candidate outlier SNPs provide valuable resources for future association studies in *C*. *gigas*.

## Introduction

The Pacific oyster (*Crassotrea gigas*), naturally distributed around Japan, China and Korea, is now an important cultivated oyster species worldwide [1]. Many countries has started introducing *C*. *gigas* since 1940s, mainly because of its rapid growth rate, high disease resistance and strong environmental adaptability [2]. The *C*. *gigas* is one of the most popular oyster species in China, and its main places of production are Shandong and Liaoning provinces [3, 4]. In 2012, China produced 3.94 million tons of oysters with *C*. *gigas* as one of the most dominant species [4, 5]. However, the broodstock used today remains largely unselected and thus *C*. *gigas* has gained little from heredity improvement by selective breeding [6].

Several selective breeding projects have been launched in couple of countries and obtained encouraging results [7–9]. With the aim of improving the productivity traits of *C*. *gigas*, a breeding program selected for fast growth has also been initiated in China. The first generation of selection was carried out by mass selection on three cultured stocks from China, Japan and Korea in 2007 and the average improvement (increase in shell height) was 10% [4]. An increase in growth rate is detected over the successive six-generation selection, but little is known about the effects of strong directional selection on genetic level in the process of cultivation. The main focus of selected lines is on inbreeding and the decrease of genetic diversity [10, 11]. Yu and Guo [11] detected rare alleles of the 4 selected strains decreased significantly compared with a wild population in Eastern oyster (*Crassostrea virginica*). Moreover, Cruz et al. [12] found selected strains through breeding programs tended to lose genetic diversity compared with wild populations in Pacific white shrimp (*Litopenaeus vannamei*). The reduction of genetic diversity in a population may possibly reduce disease resistance and decrease adaptability to environmental changes [13, 14]. Therefore, it is crucial to survey the genetic diversity within or between selected lines and wild populations for successful hatchery management of *C*. *gigas*.

In recent years, the single nucleotide polymorphisms (SNPs) and simple sequence repeats (SSRs) developed from EST sequences have been extensively used in population genetic studies. Microsatellites have been chosen in many studies for their codominant inheritance and high allelic variability [15]. However, SNP genotyping results can be compared across different platforms and laboratories more easily than microsatellite data, therefore facilitating the integration and interpretation of genotyping data across different databases. Moreover, mutations resulting in some SNPs can be responsible for an adaptive phenotype or the direct target of selection and have been shown to be correlated with economic traits in many aquatic animals [16–18]. Therefore, SNP markers offer a valuable chance to explore the genetic basis of phenotypic variation during the selective breeding program in China.

In the present study, we used 103 SNP markers to evaluate the genetic diversity level, assess the genetic differentiation, and identify putative loci under selection in three sixth-generation selected lines and four wild populations of *C*. *gigas*. The main goal is to demonstrate the effects of cultivation on genetic diversity in the selective breeding program of *C*. *gigas*, and to identify candidate SNPs under selection through the use of outlier analysis methods.

## Materials and Methods

### Ethics Statement

The *C*. *gigas* does not belong to endangered species and the sample locations are not in protection.

### Oyster Collections and DNA Extraction

**Fig 1:**
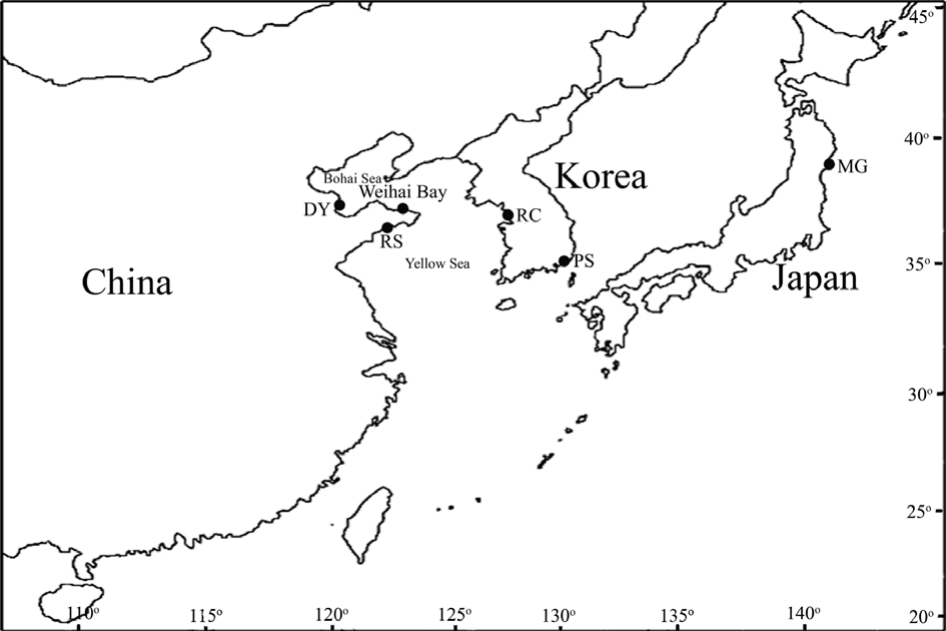
Approximate location of sampling sites shown with shaded circles. Populations are marked by abbreviations that correspond to Table 1.

Three selected lines and four wild populations of *C*. *gigas* were surveyed in our study. Collection details for the 7 *C*. *gigas* populations were shown in Fig 1 and Table 1. The cultivated oysters were randomly selected from three sixth-generation selected lines. In 2007, Pacific oysters from three cultivated populations in Pusan, South Korea (Stock K, 35.1°N, 129.1°E), Onagawa Bay, Miyagi Prefecture, Japan (Stock J, 38.3°N, 141.3°E), and Rushan, Shandong, China (Stock C, 36.4°N, 121.3°E) were used to establish selected lines for fast growth [4]. In each line, 30–60 pairs of these individuals from the top end of the shell height distribution were used as broodstock oysters for the next generation [19]. In July 2012, 91 (50 ♀×41 ♂) and 107 (59 ♀×48 ♂) individuals from the fifth-generation selected lines of Japan and Korea (JS5 and KS5) were selected as parental oysters for the sixth-generation selected lines of Japan and Korea (JS6 and KS6), respectively. In July 2013, 85 (50 ♀×35 ♂) individuals in the fifth-generation selected lines of China (CS5) were selected as parental oysters for the sixth-generation selected lines of China (CS6). Fertilization was conducted at a ratio of 50 sperm per egg with 10^7^ oocytes for each female [4, 19]. Oysters were cultivated in Weihai Bay, Shandong, China (37.3°N, 122.1°E). One-year-old oysters of the three sixth-generation selected lines (CS6, 48 individuals; JS6, 56 individuals and KS6, 48 individuals) were randomly selected for the analysis. The wild oysters were sampled from four locations: Rushan, Shandong Province, China (RS; 36.4°N, 121.3°E; 48 individuals; shell length, 3.45±0.48 cm, shell height, 5.63±0.71 cm, shell width, 1.87±0.35 cm); Dongying, Shandong Province, China (DY; 37.6°N, 119.0°E; 48 individuals; shell length, 3.93±0.68 cm, shell height, 4.96±0.90cm, shell width, 1.67±0.39 cm); Miyagi Prefecture, Japan (MG; 38.3°N, 141.3°E; 40 individuals; shell length, 4.22±0.78 cm, shell height, 7.32±0.91 cm, shell width, 2.01±0.37 cm) and Inchon, Korea (RC; 37.4°N, 126.6°E; 48 individuals; shell length, 3.62±0.78 cm, shell height, 5.73±0.98 cm, shell width, 1.63±0.39 cm). The adductor muscle was sampled and kept at −80°C. DNA was extracted using the phenol–chloroform method with a modification [20].

**Table 1:** Sample information for the Pacific oyster *Crassostrea gigas*.

Source	Abbreviation	Sample type (size)	Geographical coordinates
**Rushan, Shandong, China**	Stock C, RS	Base stock, wild population (48)	36.4°N, 121.3°E
**Onagawa Bay, Miyagi Prefecture, Japan**	Stock J, MG	Base stock, wild population (40)	38.3°N, 141.3°E
**Pusan, South Korea**	Stock K/PS	Base stock	35.1°N, 129.1°E
**Dongying, Shandong, China**	DY	Wild population (48)	37.6°N, 119.0°E
**Inchon, Korea**	RC	Wild population (48)	37.4°N, 126.6°E
**Weihai Bay, Shandong, China**	CS6, JS6, KS6	Selected lines (48, 56, 48)	37.3°N, 122.1°E

### SNP Genotyping

A total of 103 SNP markers (from CgSNP4 to CgSNP457) were used in the study [21, 22]. SNPs were genotyped using the high resolution melting (HRM) method on the LightCycler 480 real-time PCR machine (Roche Diagnostics, Burgess Hill, UK) according to the procedure described by Zhong et al. [21]. The 10-μl mixture included 0.25 U Taq DNA polymerase (Takara, Dalian, China), 10 × PCR buffer, 0.2 mM dNTP mix, 0.2 μM of each primer set, 1.5 mM MgCl_2_, 5 μM SYTO9 (Invitrogen Foster City, CA, USA) and 10 ng DNA [21]. The PCR cycling conditions were as follows: an activation step at 95°C, 5 min; 45–50 cycles of 95°C, 20 s; a touch down of 68°C to 58°C, 20 s (0.5°C /cycle) and extension for 72°C, 20 s. Before melting, the amplicons were heated at 95°C for 60s, and then cooled at 40°C for 60s. Melting curves were produced by heating amplicons from 60°C to 90°C [21].

### Data Analysis

The observed number of alleles (*Na*), effect number of alleles (*Ne*), shannon's information index (*I*), observed heterozygosity (*Ho*), expected heterozygosity (*He*), minor allele frequency (*MAF*), inbreeding coefficients (*Fis*) and chi-square analysis of Hardy–Weinberg equilibrium (*HWE*) were evaluated by POPGENE 1.32 [23]. All comparable statistics (*Na*, *Ne*, *I*, *Ho* and *He*) were calculated between wild populations and selected lines using Mann-Whitney U-Test [24] implemented with SPSS 16.0.

Pairwise *Fst* values were calculated with Arlequin 3.5.1.3 [25]. *Fst* values were calculated for significance using 10000 permutations. Gene flow among wild populations was calculated following the formula [26]:

$$Nm=\frac{\left(1-Fst\right)}{4Fst}.$$

The Nei’s genetic distance [27] was also estimated by POPGENE 1.32. The unweighted-pair-group method with arithmetic-mean (UPGMA) tree was constructed by POPTREE2 [28]. Bootstrap analyses were conducted with 1000 replicates.

The population genetic structure analysis was estimated by Structure 2.3.4 with a model-based Bayesian procedure [29, 30]. The analysis was conducted using a burn-in period of 10^5^ iterations and a run length of 10^6^ MCMC replications. The number of clusters (K) was set from 1 to 10 with three replicates. The most probable K value was evaluated by STRUCTURE HARVESTER [31, 32].

To explore candidate SNPs under selection, *Fst*-based outlier tests were conducted using two island models including a finite island model (FDIST approach) and a hierarchical island model implemented in Arlequin 3.5.1.3 [25, 33], and Bayesian likelihood approach implemented in BayeScan [34]. To detect additional evidence of selection, lnRH tests were also conducted in population pairwise comparisons.

The outlier analysis was implemented at two levels. (1) An overall analysis encompassed all populations under the hierarchical island models. Three groups were set (CS6+RS+DY+RC vs. JS6+MG vs. KS6) based on the Structure result. The analysis was conducted using 50000 coalescent simulations with 10 groups of 100 demes. (2) An analysis was conducted between selected lines and wild populations from which the selected lines originated (CS6 and RS; JS6 and MG) under a finite island-model. We conducted 50000 coalescent simulations with 100 demes to generate the joint distribution of *Fst* versus heterozygosity. Loci which were outside the 99% confidence intervals were treated as outliers potentially under selection.

The outlier tests at the two levels were also calculated with the BayeScan software. The analyses were estimated using a pilot run of 20, a burn-in of 50000 with 100000 iterations each, a sample size of 5000, a thinning interval of 10, and an FDR of 0.05. Loci with log_10_ values of the posterior odds (PO) >0.5 and 2.0 were regarded as candidate SNPs under selection with substantial and decisive evidence [35].

The lnRH test was applied to estimate the ratio of gene diversity (heterozygosity) of all loci in the population pairwise comparisons as follows:

$$\mathrm{lnRH}=\ln\left(\frac{{\left(\frac{1}{1-H_{\mathrm{pop}1}}\right)}^{2}-1}{{\left(\frac{1}{1-H_{\mathrm{pop}2}}\right)}^{2}-1}\right)$$

where H_pop1_ and H_pop2_ denote expected heterozygosity for population 1 and population 2 [36]. The lnRH values were standardized for each population comparisons and therefore the standardized distributions had a mean of zero and a standard deviation of one [37]. Those SNPs fell out of the 95% interval were taken as candidate loci under selection.

Sequence annotation was conducted by BLASTx software in NCBI database (http://www.ncbi.nlm.nih.gov/) and OysterBase (http://www.oysterdb.com), and the critical *E* value was set as 1.0×10^−6^. The NCBI ORF finder (http://www.ncbi.nlm.nih.gov/gorf/gorf.html) was used to distinguish synonymous SNPs, non-synonymous SNPs or SNPs from untranslated regions (UTRs). The putative function of genes was identified by using the Gene Ontology (GO) annotation by mining the Swiss-Prot database (http://us.expasy.org/sprot/sprot-top.html) and OysterBase.

## Results

### Genetic Diversity

Genetic variability indices for the four wild populations and three selected lines of *C*. *gigas* were shown in Table 2. For all the genetic diversity parameters, the selected lines had lower values (mean *Na*, *Ne*, *I*, *Ho* and *He*) than the wild populations, but no significant loss of *Ne*, *I* or *He* were detected (*P* > 0.05). The observed heterozygosities ranged from 0.2703 to 0.2939 with a mean of 0.2806 in the wild populations, and ranged from 0.2479 to 0.2733 with a mean of 0.2599 in the selected lines. There is a significant reduction of observed heterozygosities in the selected lines in contrast with the wild populations (*P* = 0.05). The observed number of alleles per locus varied from 1.9417 to 1.9709 in the selected lines, and varied from 1.9806 to 2.000 in the wild populations. There was a significant reduction of the observed number of alleles per locus in the selected lines compared with the wild populations (*P* < 0.05).

**Table 2:** Genetic diversity parameters of selected lines and wild populations.

Population	*Na*	*Ne*	*I*	*Ho*	*He*
**CS6**	1.9709±0.1690	1.5031±0.3206	0.4599±0.2034	0.2733±0.1766	0.3053±0.1585
**JS6**	1.9515±0.2160	1.4780±0.3472	0.4332±0.2247	0.2586±0.1846	0.2867±0.1729
**KS6**	1.9417±0.2354	1.4539±0.3464	0.4146±0.2346	0.2479±0.1969	0.2743±0.1772
**RS**	1.9903±0.0985	1.5138±0.3033	0.4774±0.1748	0.2939±0.1613	0.3154±0.1416
**MG**	2.000±0.0000	1.5362±0.3025	0.4892±0.1742	0.2801±0.1576	0.3261±0.1426
**RC**	1.9806±0.1387	1.5052±0.3206	0.4639±0.1945	0.2703±0.1637	0.3070±0.1548
**DY**	1.9806±0.1387	1.5012±0.3032	0.4664±0.1879	0.2780±0.1601	0.3083±0.1481
**Selected**	1.9547±0.2068	1.4783±0.3381	0.4359±0.2209	0.2599±0.1860	0.2888±0.1695
**Wild**	1.9879±0.0940	1.5141±0.3074	0.4742±0.1829	0.2806±0.1607	0.3142±0.1468
**Total**	1.9713±0.1504	1.4962±0.3228	0.4551±0.2019	0.2703±0.1734	0.3015±0.1582

Note: *Na*, observed number of alleles; *Ne*, effective number of alleles; *I*, shannon's information index; *Ho*, observed heterozygosity; *He*, expected heterozygosity.

Information of the 103 SNPs evaluated from the three selected lines and four wild populations were summarized in S1 Table. All loci had two alleles in the 7 populations except that 19 loci had only one allele in one or more populations, including 3 loci (CgSNP252, CgSNP265 and CgSNP283) in CS6, 5 loci (CgSNP149, CgSNP176, CgSNP254, CgSNP265 and CgSNP305) in JS6, 6 loci (CgSNP14, CgSNP140, CgSNP176, CgSNP187, CgSNP252 and CgSNP319) in KS6, 1 locus (CgSNP283) in RS, 2 loci (CgSNP254 and CgSNP309) in RC and 2 loci (CgSNP176 and CgSNP283) in DY. A total of 83 of the 721 single-locus exact tests showed significant departures from *HWE* after sequential Bonferroni correction (*P* < 0.05/103), and all loci showed heterozygote deficiencies except for CgSNP261 in CS6 and JS6, CgSNP36 in KS6 and MG, and CgSNP402 in RS.

### Genetic Differentiation

Most pairwise *Fst* values were significant (*P*<0.001), except for that estimated between wild populations RS and RC. The lowest value was observed between RS and RC (*Fst* = 0.00689), however the highest value was detected between JS6 and KS6 (*Fst* = 0.11737) (Table 3). Within the selected lines, the values varied from 0.06941 (KS6 and CS6) to 0.11737 (JS6 and KS6). Within the wild populations, the values ranged from 0.00689 (RS and RC) to 0.07046 (RC and MG). Moreover, gene flow among the wild populations ranged from 3.29811 (MG and RC) to 36.03447 (RS and RC).

**Table 3:** Pairwise *Fst* values (lower diagonal) and Nei’s genetic distance (upper diagonal) among seven populations.

	CS6	JS6	KS6	RS	DY	MG	RC
**CS6**	0	0.0407	0.0349	0.0190	0.0323	0.0451	0.0193
**JS6**	0.07944	0	0.0571	0.0407	0.0357	0.0226	0.0443
**KS6**	0.06941	0.11737	0	0.0382	0.0542	0.0552	0.0346
**RS**	0.03067	0.07742	0.07460	0	0.0160	0.0413	0.0083
**DY**	0.05764	0.06847	0.10685	0.02380	0	0.0354	0.0191
**MG**	0.07851	0.04069	0.10513	0.06930	0.06006	0	0.0408
**RC**	0.03149	0.08571	0.06802	0.00689	0.03035	0.07046	0

**Fig 2:**
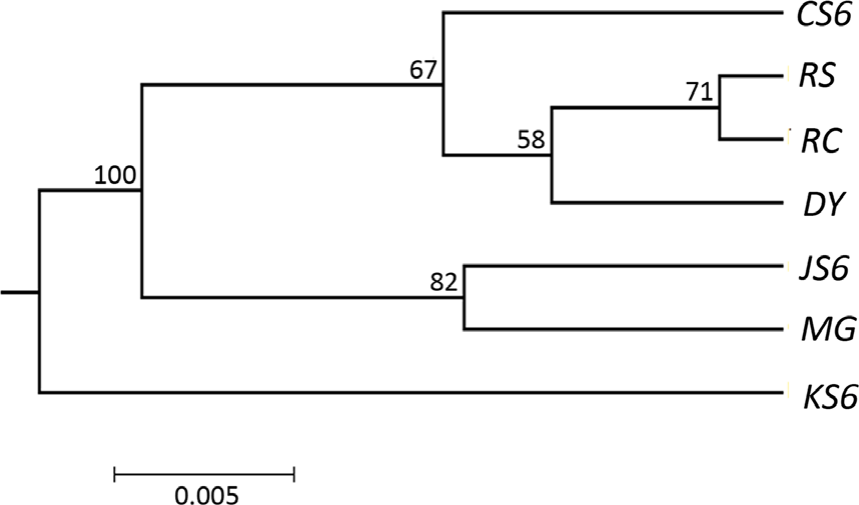
Phylogenetic tree of four wild populations (RS, DY, RC and MG) and three selected lines (CS6, KS6 and JS6) using the unweighted pair-group method with arithmetic mean (UPGMA) based on Nei’s genetic distance derived from 103 SNPs. Numbers above branches indicate bootstrap values.

The pairwise Nei's genetic distances among populations were also shown in Table 3. The lowest value was observed between RS and RC (0.0083), while the highest value was detected between KS6 and JS6 (0.0571). The UPGMA tree was constructed based on pairwise genetic distance (Fig 2). The UPGMA tree separated the 7 populations into two clusters. The KS6 originated from Korea belonged to one cluster while the other cluster included the other 6 *C*. *gigas* populations. Moreover, this cluster was further divided into 2 subgroups; the first subgroup contained JS6 and MG originated from Japan, and the second subgroup contained CS6, RS and DY originated from China, and RC from Korea.

### Genetic Structure

The genetic structure test indicated that the most likely number of genetic groups was K = 3. The first group consisted of CS6, RS and DY originated from China, and RC from Korea. The second group consisted of JS6 and MG originated from Japan. Moreover, the selected line KS6 originated from Korea constituted the third genetic group (S1 Fig).

### Outlier SNPs

**Fig 3:**
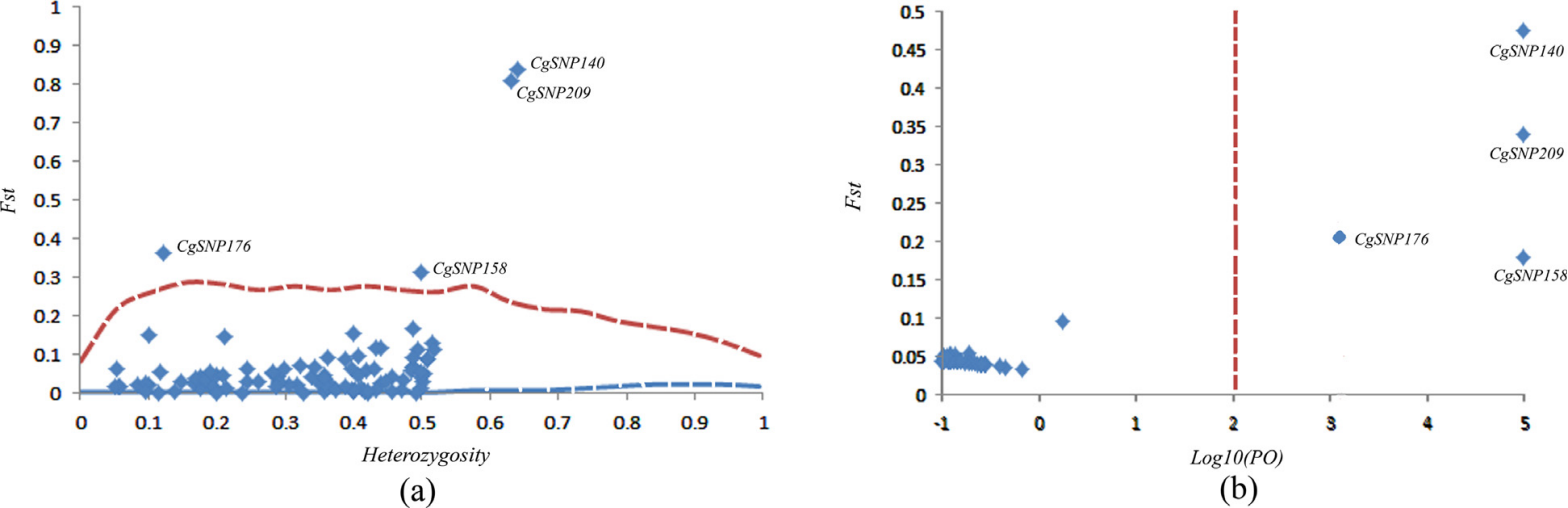
Results of two outlier tests in all populations. Locus names of putative outliers potentially affected by selection are indicated. (a) Hierarchical island model: empirical distribution of *Fst* against heterozygosity. The upper and lower lines are the 99% confidence intervals. (b) BayeScan: *Fst* estimates plotted against log_10_ of the posterior odds (PO). The dashed lines correspond to the posterior odds 100 (log_10_(PO) = 2).

Both the hierarchical island model and Bayesian likelihood approach revealed 4 loci (CgSNP140, CgSNP158, CgSNP176 and CgSNP209) under selection across all populations (Table 4; Fig 3). To detect specific footprint of artificial selection at regional level, we conducted pairwise tests between selected lines and wild populations from which the selected lines originated. The Fdist method revealed 5 candidate SNPs under selection, including 3 loci (CgSNP40, CgSNP225 and CgSNP236) for populations originated from China (CS6+RS) and 2 loci (CgSNP176 and CgSNP197) for populations originated from Japan (JS6+MG) (Table 4). The same two SNPs (CgSNP 225 and CgSNP 176) were also detected by BayeScan. The lnRH test deteced 12 outlier SNPs, including 5 SNPs (CgSNP23, CgSNP82, CgSNP83, CgSNP225 and CgSNP236) for CS6-RS comparison, 6 SNPs (CgSNP33, CgSNP140, CgSNP194, CgSNP283, CgSNP375 and CgSNP397) for JS6-MG comparison and one common SNP (CgSNP 309) for the two population comparisons. Totally, 17 candidate outlier SNPs were found under selection using Arlequin, BayeScan and lnRH tests. Among the 17 SNPs, 16 were detected in the coding region, and 1 in the UTR. Among the 16 SNPs located in the coding region, 11 SNPs were synonymous and 5 nonsynonymous. Nine SNPs could be annotated by BLASTx software. The CGI_10015432, CGI_10017873, NADH dehydrogenase [ubiquinone] iron-sulfur protein 2 and Collagen alpha-5(VI) chain were involved in G-protein coupled receptor protein signaling pathway, lipid transport, oxidation-reduction process and lipoprotein metabolic process, however, the putative function of other five proteins (CGI_10028477, flotillin 2, CGI_10002462, UPF0451 protein C17 or f61-like protein and CGI_10010736) could not be identified using GO searches.

**Table 4:** Outlier SNPs detected using Arlequin, BayeScan and lnRH tests.

	Arlequin				BayeScan				LnRH test				
	Locus	*Ho*	*Fst*	*P*	Log_10_(*PO*)	*Fst*	*Probi*	*Alpha*	LnRH	Type		Annotation	
**All populations**	CgSNP140	0.640	0.837	0.000	1000	0.476	1.000	3.268		S (Ala)		CGI_10015432	
	CgSNP158	0.500	0.311	0.002	1000	0.179	1.000	1.566		UTR		Unknown	
	CgSNP176	0.121	0.363	0.002	3.097	0.205	0.999	1.732		N (Cys/Tyr)		CGI_10028477	
	CgSNP209	0.631	0.809	0.000	1000	0.341	1.000	2.588		S (Glu)		Unknown	
**CS6+RS**	CgSNP23	-	-	-	-	-	-	-	-2.258	N (The/Ala)		Unknown	
	CgSNP40	0.570	0.239	0.008	-	-	-	-	-	S (Arg)		Unknown	
	CgSNP82	-	-	-	-	-	-	-	2.265	S (Ser)		Unknown	
	CgSNP83	-	-	-	-	-	-	-	2.265	S (Met)		Unknown	
	CgSNP225	0.328	0.266	0.001	0.628	0.110	0.809	1.124	-3.628	S (Leu)		Hypothetical protein flotillin 2	
	CgSNP236	0.379	0.250	0.003	-	-	-	-	2.947	N (Pro-Thr)		Hypothetical protein CGI_10002462	
	CgSNP309	-	-	-	-	-	-	-	-2.084	S (Asn)		Collagen alpha-5(VI) chain	
**JS6+MG**	CgSNP33	-	-	-	-	-	-	-	-3.137	N (Gln/Arg)		Unknown	
	CgSNP140	-	-	-	-	-	-	-	-2.092	S (Ala)		CGI_10015432	
	CgSNP176	0.415	0.497	0.000	3.398	0.241	1.000	1.995	Not fit	N (Cys/Tyr)		CGI_10028477	
	CgSNP194	-	-	-	-	-	-	-	2.150	S (Lys)		Unknown	
	CgSNP197	0.438	0.230	0.007	-	-	-	-	-	N (Glu/Lys)		CGI_10017873	
	CgSNP283	-	-	-	-	-	-	-	-2.249	S (Ser)		NADH dehydrogenase [ubiquinone] iron-sulfur protein 2	
	CgSNP309	-	-	-	-	-	-	-	-2.370	S (Asn)		Collagen alpha-5(VI) chain	
	CgSNP375	-	-	-	-	-	-	-	-2.928	S (His)		UPF0451 protein C17or f61-like protein	
	CgSNP397	-	-	-	-	-	-	-	-2.092	S (Ser)		Hypothetical protein CGI_10010736	

Note: *Ho*, observed heterozygosity; *PO*, posterior odds; *Alpha*, positive values indicate positive selection, while negative values indicate putative balancing selection; *Probi*, probability; *Not fit*, CgSNP176 was monomorphic in JS6, thus not fit for the lnRH test; *S*, synonymous; *N*, non-synonymous; *UTR*, untranslated region.

## Discussion

### Genetic Diversity

Maintenance of genetic variation is known to be important for long-term survival of populations because the level of variation determines their adaptability to environmental changes [38, 39]. Heterozygosity in many instances is considered as the primary parameter to reflect overall genetic variability of populations [40, 41]. In our study, the selected lines showed a significant reduction in the observed heterozygosity contrasted with the wild populations. Similar results have been detected in cultivated Atlantic salmon (*Salmo salar*) and Pacific abalone (*Haliotis discus hannai*) populations [42, 43].

Many researches have indicated loss of alleles is often more easily observed than decrease of heterozygosity in cultivated or selected populations [10, 11, 44]. Yu and Guo [11] detected the decrease in allele number was significant for selected lines NEH (by 36.8%) and CTS (by 35.1%) contrasted with the wild population of *C*. *virginica*. Wang et al. [45] found the cultured populations lost 9 of the 45 alleles (20%) compared with the wild population of bay scallop (*Argopecten irradians*). Compared with wild populations, 20% to 48% fewer alleles were detected in farmed salmon strains from Ireland and Norway [46]. In our study, the selected lines exhibited a significant reduction in observed number of alleles in contrast with the wild populations (*P*<0.05). Moreover, 0.98% (2/205) and 2.43% (5/206) fewer alleles were found in selected lines CS6 and JS6 than their wild progenitors (RS and MG), respectively. The CS6 stock seemed to be missing two rare alleles at 2 loci (CgSNP252 and CgSNP265) compared with RS. No individuals with the rare allele of the CgSNP265 found rarely in RS, were sampled from CS6. There was a possibility that this rare allele existed in CS6, but was not sampled in the population. As this rare allele frequency was 0.0521 in RS, the probability of completely missing this rare allele in the CS6 (48 samples) should be estimated as 0.9479^96^ = 0.0059. Therefore, it is most likely that the CS6 has indeed lost this rare allele at the CgSNP265. Similarly, the JS6 may have indeed lost rare alleles at 4 loci (CgSNP149, CgSNP176, CgSNP254 and CgSNP305). Taken together, we can make a conclusion that the selected lines through breeding programs tended to lose genetic diversity compared with the wild populations. The decrease of genetic diversity may result from genetic drift, bottlenecks, and inbreeding caused by a reduced effective population size.

Significant deviations from *HWE* were detected both in selected lines and wild populations. In selected lines, heterozygote deficiency may be mainly because of a limited number of founders and artificial selection [47]. In the wild populations, heterozygote deficiency may be cause by Wahlund effect and natural selection. As heterozygotes with null alleles may be taken as homozygotes, null alleles may also account for the heterozygote deficiency.

### Genetic Differentiation

The UPGMA analysis indicated that the selected lines and wild populations were not separated into two groups and even the wild populations and selected lines from the same origin country were culstered into the same subgroup except for KS6 and RC, suggesting there was no clear division between the wild populations and selected lines. The result is also supported by the Structure analysis.

Within the selected lines, moderate genetic differentiation (0.05<*Fst*<0.15) was detected. The UPGMA analysis also demonstrated the three selected lines fell into three groups. The significant genetic differentiation among the three selected lines may result from three different founder populations, thus providing the genetic basis for the establishment of selected lines for faster growth using three *C*. *gigas* stocks from China (Rushan), Japan (Miyagi Prefecture) and Korea (Pusan).

Within the wild populations, slight genetic differentiations (*Fst*<0.05) were detected among the three populations RS, DY and RC. The *Nm* values among these populations ranged from 7.98723 (RC and DY) to 36.03447 (RS and RC), which appeared to be sufficiently large to swamp potential for large genetic differences. Since oyster adults are sedentary, larval dispersal becomes a decisive factor affecting gene flow among wild populations. Therefore, the long pelagic larval phase (14–21 days) and high fecundity (10 to 50×10^6^ oocytes per female) of the Pacific oyster may account for the relatively large gene flow detected among RS, DY and RC [48, 49]. However, moderate genetic differentiations were found between population MG and other three populations RS, DY and RC, suggesting potential barriers to gene exchange may exist. Many studies showed a significant relationship between gene flow and geographic distance, suggesting that a pattern of isolation by distance (IBD) explained a great proportion of gene flow in *Mactra chinensis* and *Crassostrea ariakensis* [50, 51]. The IBD pattern indicates long geographic distance can restrict migration among wild populations, which may explain the restricted gene flow between population MG and other three populations RS, DY and RC. Overall, the genetic relationship among the four wild populations could be visualized using the UPGMA tree, which indicated that RS and RC had the closest relationship with DY being the sister group, while MG was in another clade.

### Outlier SNPs

In order to improve the productivity traits of *C*. *gigas*, successive generations of mass selection for fast growth were conducted in three *C*. *gigas* stocks from China, Japan and Korea since 2007. As there are similarities in controlled selective regimes, the selected lines may provide a valuable opportunity to study parallel evolution, which is taken as one of the most convincing manifestations of the effect of selection in driving adaptive change [52, 53]. Our study provided support for the hypothesis of parallel evolution at the DNA level as one outlier SNP (CgSNP309) was overlapped in the two wild-selected population comparisons. The parallel evolution is also shown in two ways in transcription level, one by the same DNA sequence polymorphisms with direct changes in gene expression, and the other by the different DNA sequence polymorphisms with the same downstream pathways during the cultivation [54]. Flori et al. [55] detected that different DNA sequence polymorphisms but the same physiological pathways were influenced during the cultivation in dairy cattle. The similar results were also found in rainbow trout (*Oncorhynchus mykiss*) and brook charr (*Salvelinus fontinalis*) [56, 57]. However, we can not conclude that parallel evolution at functional levels may account for the result in our study as only four outliers could be annotated with GO terms.

Four different tests (hierarchical island model, fdist island model, Bayesian likelihood approach and lnRH test) were used to detect outlier SNPs potentially under selection during the selective breeding program. These approaches rely on the rationale that selection will lead to increased genetic differentiation between populations and should exhibit a reduction or elevation in genetic variation contrasted with neutral genes. Actually, selection is not the only cause of variation changes at particular loci, reduced variation or increased differentiation can also arise from genetic drift, bottlenecks or founder events [58]. The effective population size for these selected lines is likely to have been less than 100, allowing for substantial random genetic drift over six generations.

The highly consistent results from the hierarchical island model and Bayesian likelihood approach for global outlier analyses strongly suggested that the identified outlier SNPs were subject to selection. Moreover, the same three SNPs (CgSNP140, CgSNP176 and CgSNP209) were also detected as outliers using selected lines and wild populations from which the selected lines originated (CS6, RS, JS6 and MG) (data not shown). At regional level, 3 SNPs (CgSNP225, CgSNP 236 and CgSNP 176) was detected as outlier using at least two analysis methods. Those outlier SNPs found in only one test should be considered with caution. The inconsistent results obtained by the three methods (fdist island model, Bayesian likelihood approach and lnRH test) were probably due to the different measures of variability and different assumptions [59]. Although the outlier analysis provides an encouraging result, association genetics and functional studies are ultimately required to confirm the outlier loci are involved in the artificial selection during the selective breeding program in China.

In this study, SNPs has been demonstrated to be a good marker of choice for monitoring the genetic variation in wild populations and selected lines of *C*. *gigas*. The genetic diversity analysis showed that the selected lines tended to lose genetic diversity contrasted with the wild populations during the successive six-generation selection. Moreover, the UPGMA results suggested there was no clear division between the wild populations and selected lines. In addition, a total of 17 loci were found under selection at two levels using four outlier tests. Further functional studies are needed to confirm the role of the candidate outlier SNPs during the domestication process in *C*. *gigas*.

## Supporting Information

S1 Fig Population genetic structure analysis with software Structure 2.3.4. (a) Genetic clusters obtained with three groups. Each individual is represented by one vertical line with 3 segments colored proportionally according to their belonging to a genetic group. Black lines separate individuals from different populations. (b) Graph of delta K.
(TIF)

S1 Table Summary of the statistics for 103 SNP loci in the wild and selected *Crassostrea gigas* populations. (XLS)
