# Supplementary figures and images for: Genetic Variation and Breeding Signature in Mass Selection Lines of the Pacific Oyster (Crassostrea gigas) Assessed by SNP Markers
Source: PLoS One. 2016 Mar 8;11(3):e0150868. doi: 10.1371/journal.pone.0150868 (PMC4783100; doi:10.1371/journal.pone.0150868)

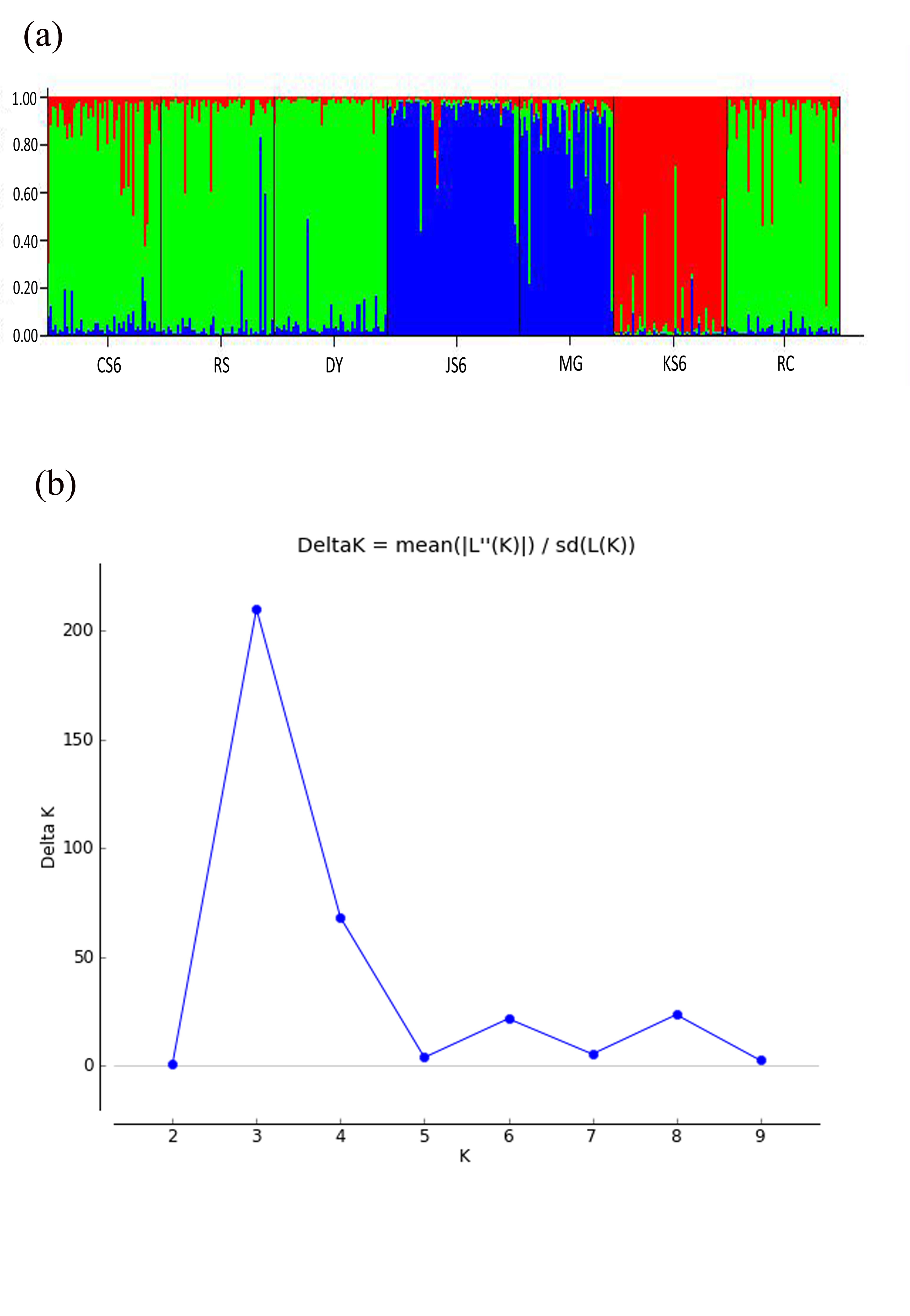

Supplement: S1 Fig — (a) Genetic clusters obtained with three groups. Each individual is represented by one vertical line with 3 segments colored proportionally according to their belonging to a genetic group. Black lines separate individuals from different populations. (b) Graph of delta K. (TIF) [file pone.0150868.s001.tif]
